# Overlapping Leigh Syndrome/Myoclonic Epilepsy With Ragged Red Fibres in an Adolescent Patient With a Mitochondrial DNA A8344G Mutation

**DOI:** 10.3389/fneur.2018.00724

**Published:** 2018-09-13

**Authors:** Cunzhou Shen, Wenbiao Xian, Hongyan Zhou, Xunhua Li, Xiuling Liang, Ling Chen

**Affiliations:** Department of Neurology, National Key Clinical Department and Key Discipline of Neurology, The First Affiliated Hospital, Sun Yat-Sen University, Guangzhou, China

**Keywords:** Leigh syndrome, MERRF, mitochondrial DNA, A8344G mutation, mitochondrial disease

## Abstract

We present the case of a 16-year-old boy with a family history of epilepsy who presented with acute respiratory failure, limb weakness, diabetes mellitus, sinus tachycardia, lactic acidosis, and pneumonia. He went on to develop cranial nerve palsy, myoclonus, generalized seizures, ataxia, recurrent pneumonia, and hypotension. Biochemical investigation revealed elevated lactate, pyruvate, and glucose levels. Cerebral magnetic resonance imaging (MRI) revealed bilateral, symmetric, high-intensity T2-weighted signals in the thalamus, brainstem, and gray matter of the spinal cord. Histochemical analyses revealed ragged red fibers (RRF) and decreased cytochrome oxidase activity. Blood and muscle-derived DNA demonstrated a high level (95% and 96%, respectively) of the m.8344A>G mutation, while almost all of his maternal relatives (*n* = 17, including his mother) carried the same point mutation. The point mutation level of his mother (who had short stature, high blood lactate levels, and epilepsy) was 77% (blood-derived DNA). Although this mutation has been identified in approximately 30 individuals with these disorders, to our knowledge, this is the first reported case of overlapping Leigh syndrome/myoclonic epilepsy with RRF in an adolescent patient, and the largest reported pedigree of mitochondrial DNA A8344G mutation.

## Introduction

Mitochondrial disease encompasses a genetically and clinically heterogeneous group of diseases caused by defects in mitochondrial oxidative phosphorylation. Such diseases, including Leigh syndrome (subacute necrotising encephalomyelopathy) and myoclonic epilepsy with ragged red fibers (MERRF), may be caused by mutations in either mitochondrial or nuclear DNA (1–3).

Leigh syndrome is most frequently characterized by the presence of focal, bilateral, and symmetric brain lesions, particularly in the basal ganglia, thalamus, and brainstem (4). Myoclonus, generalized seizures, ataxia, and the presence of ragged red fibers (RRF) upon muscular biopsy characterize MERRF (5–7). However, both Leigh syndrome and MERRF exhibit considerable clinical and genetic heterogeneity.

A8344G mutations of the mitochondrial DNA (mtDNA) are associated with Leigh syndrome, MERRF, and other diseases (8–10). Although some mtDNA mutations tend to be associated with specific clinical syndromes, the genotype-phenotype correlations are imprecise. Some individuals who harbor a pathogenic mtDNA mutation exhibit overlapping features of typical mitochondrial syndromes (11–13).

Here, we present the first reported case of overlapping Leigh syndrome/MERRF, which developed almost simultaneously, in an adolescent patient with an mtDNA A8344G mutation.

## Case report

### Case history

A 16-year-old boy with a family history of seizures (mother, cousin) exhibited typical development until the age of 14; this was the point at which he developed generalized epilepsy, which was well-controlled using valproic acid (VPA). He also had a family history of high blood lactate levels (mother) and a history of easy fatigability. At the age of 16, he developed tachypnoea and tachycardia. Serum glucose and lactate levels were elevated to 12.5 and 9.4 mmol/L, respectively. Urine tests for glucose and ketones were positive (3+ and 2+, respectively). Arterial blood gas analysis in room air revealed elevated lactate levels (9.4 mmol/L) and low pH (7.23). His glycosylated hemoglobin A1 (HbA1c) level was 14.60%. He was diagnosed with diabetes, ketoacidosis, and generalized epilepsy. However, his high lactate levels and tachycardia persisted following treatment for hyperglycaemia, fluid resuscitation, and correction of acidosis. Several days later, his tachypnoea returned, and he also developed limb weakness and external ophthalmoplegia. After 1 week, he developed severe respiratory acidosis and respiratory failure type II, for which he required intubation and artificial ventilation. Tracheotomy was performed after several failed attempts to discontinue artificial ventilation.

Upon admission, neurological examination revealed external ophthalmoplegia, mild limb weakness, and pyramidal signs. However, he exhibited no signs of myoclonus or cognitive abnormalities. Laboratory testing revealed an increase in plasma lactate (9.4 mmol/L, normal <2.3), pyruvate (D-3-hydroxybutyrate, 0.35 mmol/L, normal 0.03–0.30 mmol/L), and glucose (12.5 mmol/L, normal <7.0 mmol/L) levels. Fasting plasma insulin and 30 min and 2 h post-prandial insulin values were 112.54, 298.03, and 73.34 μU/mL, respectively. Creatine kinase levels were normal. Arterial blood gas analysis indicated severe respiratory acidosis. His mother also exhibited increase in resting and post-exercise blood lactate levels (2.7 and 9.7 mmol/L, respectively). Cerebrospinal fluid analysis revealed slight increase in intracranial pressure, normal white and red cell counts, glucose levels, and protein levels. Bacterial cultures were negative, and no abnormal cells were detected.

Electrocardiography revealed sinus tachycardia. Normal structure and ejection fraction were observed via echocardiography. Electroencephalography revealed moderate abnormalities, without spikes or sharp waves. Electromyography revealed mild axonal damage and demyelination, as well as abnormal F waves.

**Figure 1:**
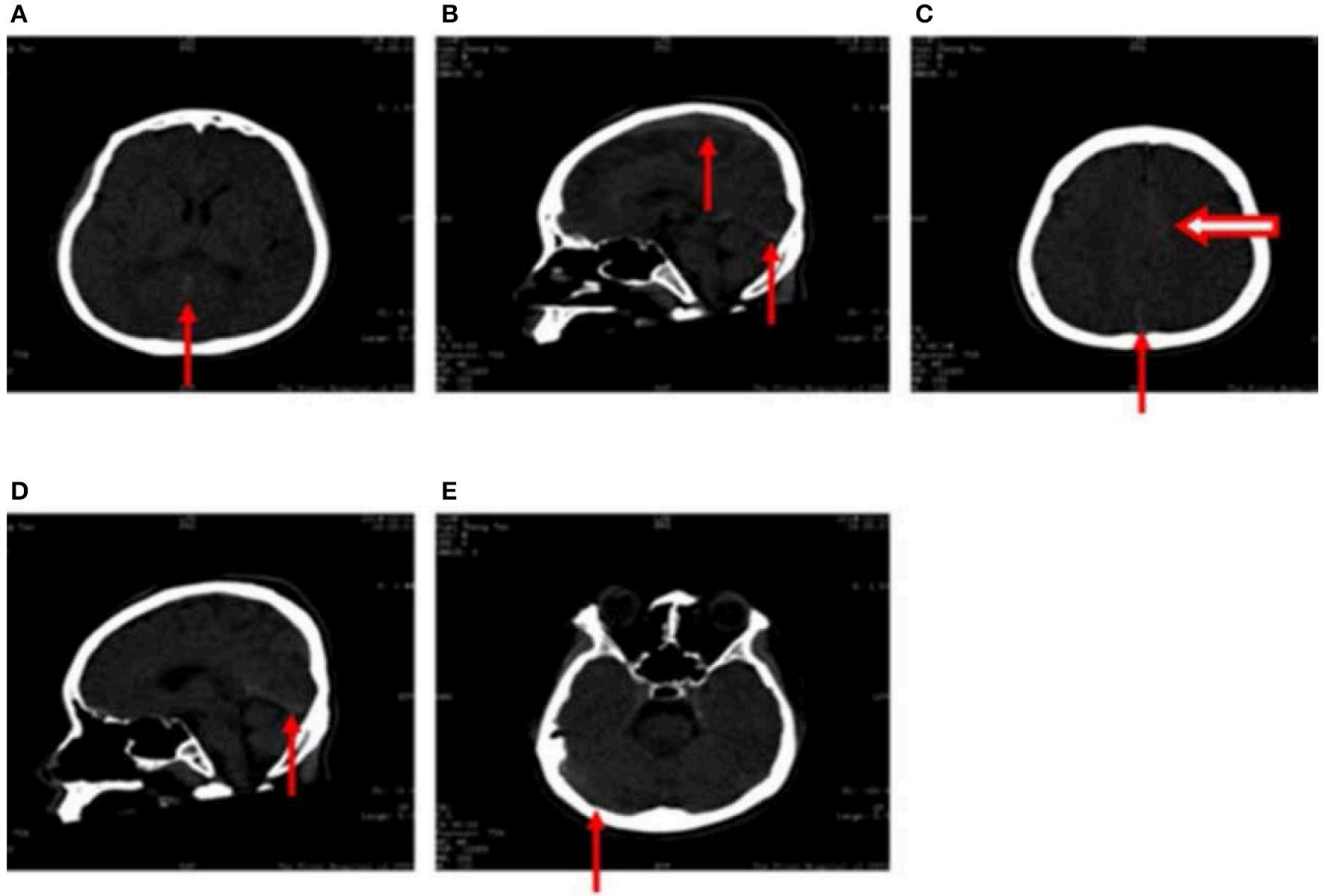
**(A–E)** Computed tomography images obtained following hospitalization show high-density signals in the great cerebral vein (arrow in **A**), sagittal sinus (arrow in **B,D**), torcular herophili (arrow in **C**), transverse sinus (arrow in **E**) (thrombus formation was not excluded). High-density signals were also observed in the cerebral falx (hollow arrow in **C**), leading us to consider subarachnoid hemorrhage.

High-density computed tomography (CT) signals were observed in the great cerebral vein (arrow in Figure 1A), sagittal sinus (arrow in Figures 1B,D), torcular herophili (arrow in Figure 1C), and transverse sinus (arrow in Figure 1E). Thrombus formation had not been excluded at this stage. High-density CT signals were also observed in the cerebral falx (hollow arrow in Figure 1C), which was suggestive of subarachnoid hemorrhage. However, results of CT angiography taken 2 days after fluid resuscitation were normal.

**Figure 2:**
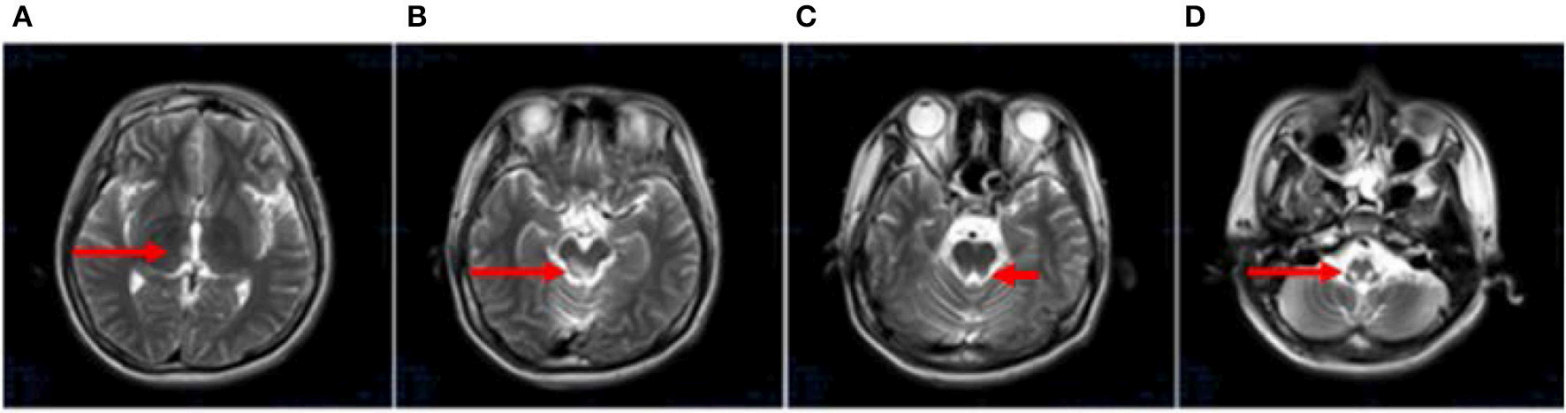
**(A–D)** Axial, T2-weighted MRI at 1 month following hospitalization revealed high signal intensity in the lateral ventricle, periaqueductal gray matter (bilateral hypothalamus and midbrain tegmentum, arrow in **A,B**), and medullary tegmentum (arrow in **D**). There were no evident lesions in the tegmentum of pons at this stage (small arrow in **C**).

**Figure 3:**
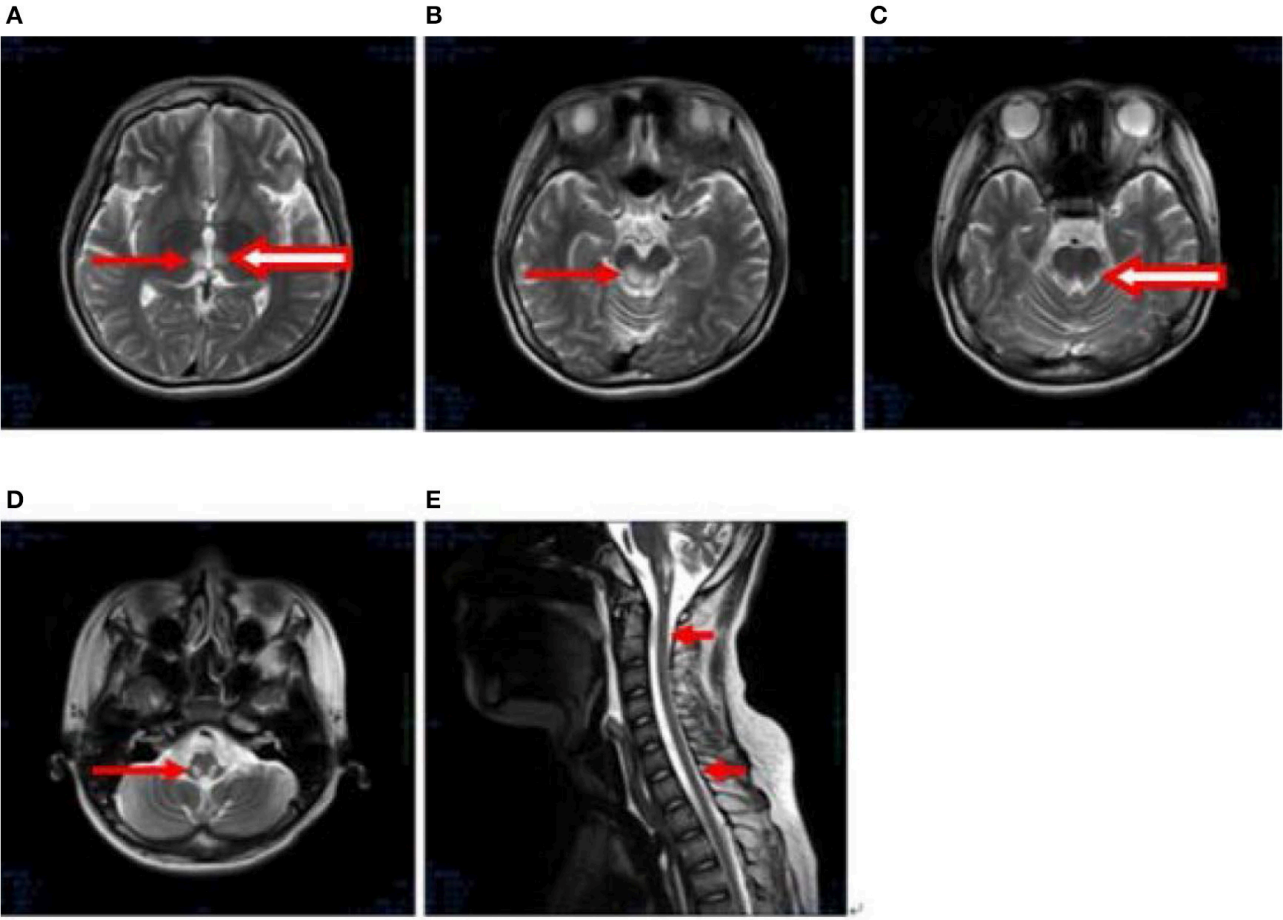
**(A–E)** Axial, T2-weighted MRI at 2 months following hospitalization revealed hyperintensities in the bilateral thalamus (arrow and hollow arrow in **A**), tegmental area of the midbrain (arrow in **B**), pons (hollow arrow in **C**), and medulla (arrow in **D**). The thalamic lesions had increased in size, and new lesions had emerged (hollow arrow in **A,C**). There were no evident lesions in the cervical and thoracic spinal cord at this stage (small arrows in **E**).

**Figure 4:**
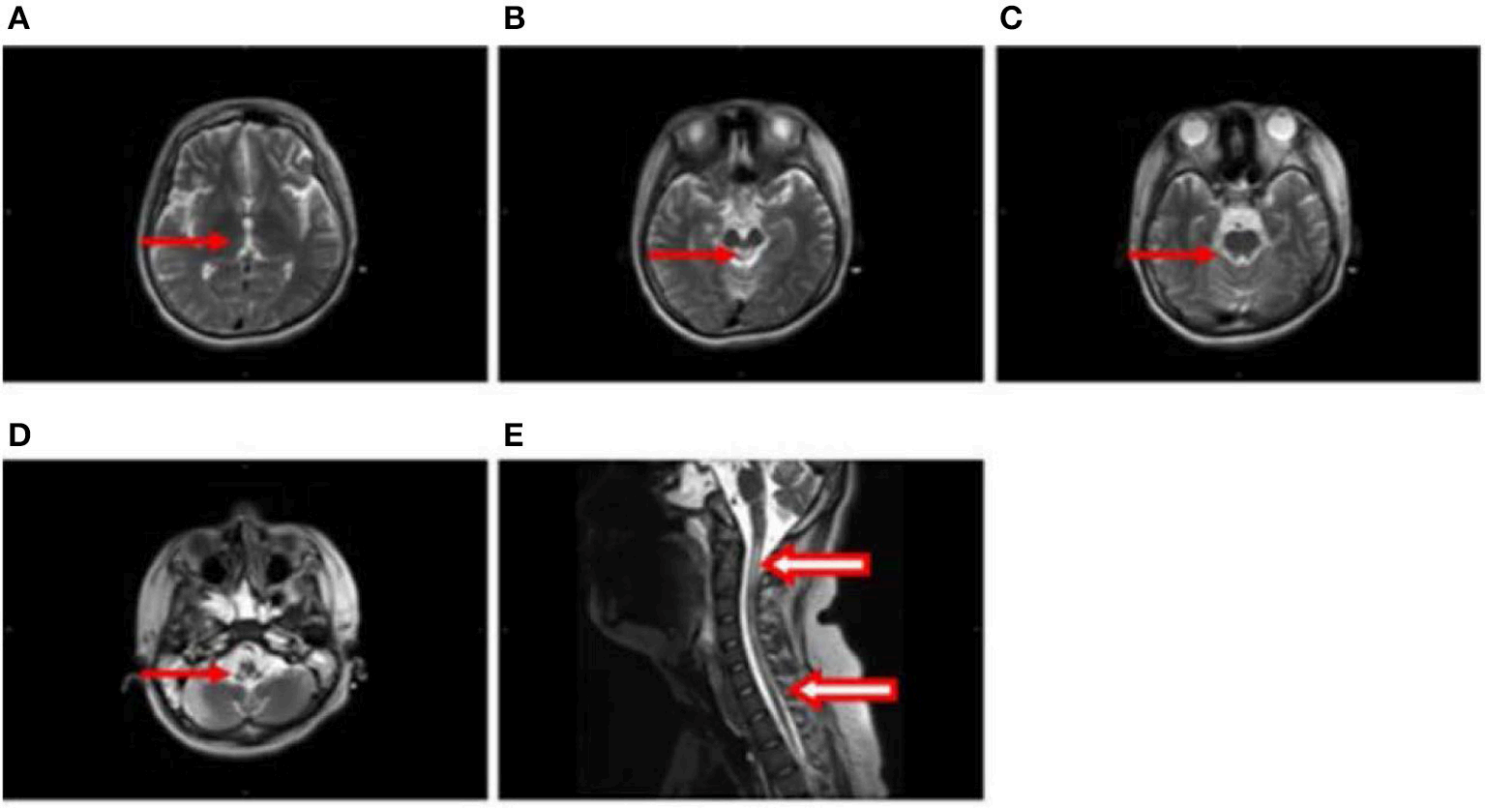
**(A–E)** Axial and sagittal T2-weighted MRI at 7 months following hospitalization revealed hyperintensities in the bilateral thalamus (arrow in **A**), tegmental area of the midbrain (arrow in **B**), pons (arrow in **C**), medulla (arrow in **D**), and the cervical and thoracic spinal cord (new lesions, hollow arrow in **E**). The previously identified lesions had decreased in size because of atrophy.

**Figure 5:**
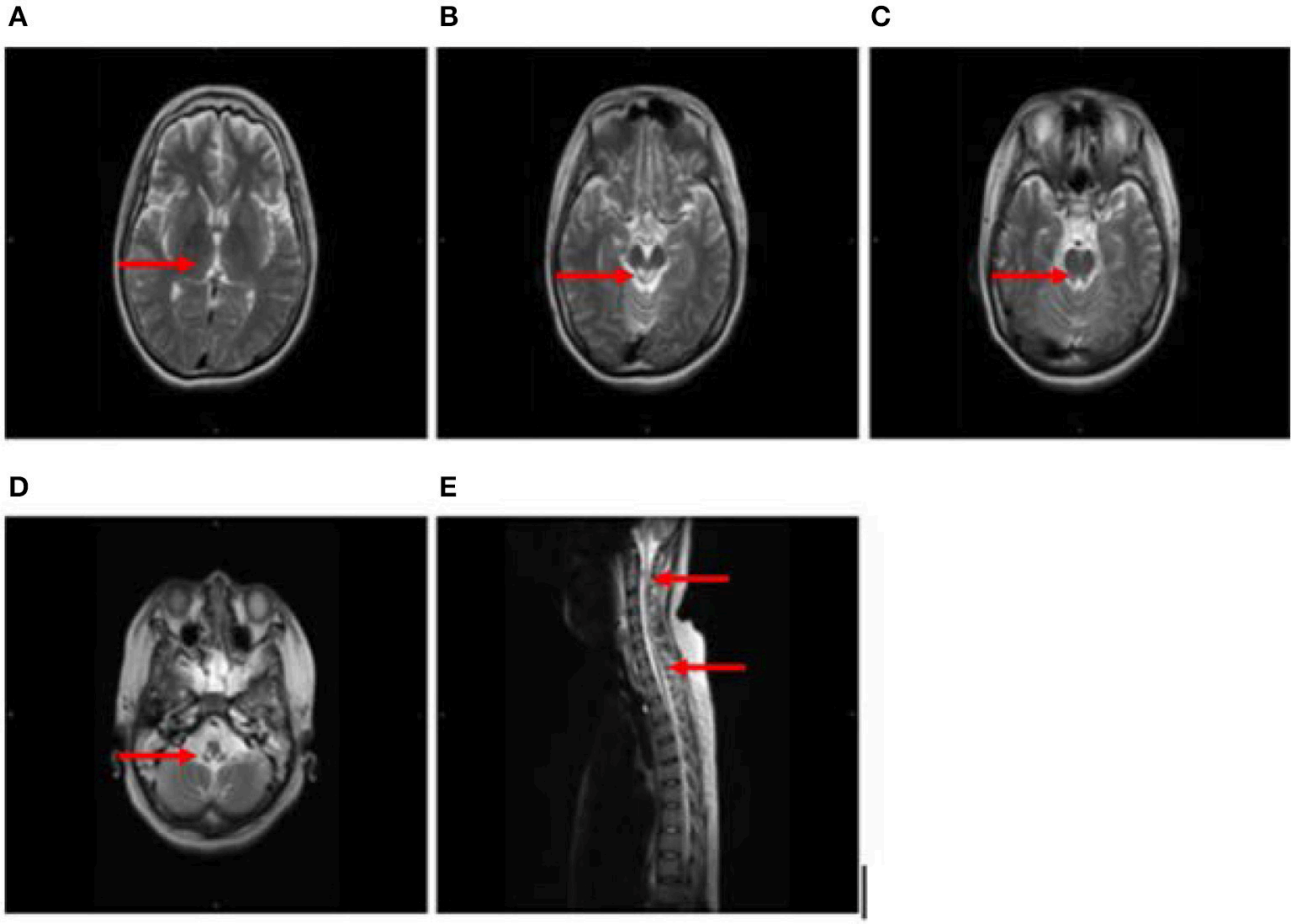
**(A–E)** T2-weighted MRI results at 8 months following hospitalization were similar to those obtained 1 month earlier (arrow in **A–E**).

After 1 month, T2-weighted magnetic resonance imaging (MRI) (Figure 2) revealed areas of high signal intensity in the lateral ventricle, periaqueductal gray matter (bilateral hypothalamus and midbrain tegmentum, arrow in Figures 2A,B), and medullary tegmentum (arrow in Figure 2D). There were no evident lesions in the tegmentum of pons at this stage (small arrow in Figure 2C). After 2 months, T2-weighted MRI (Figure 3) revealed hyperintensities in the bilateral thalamus (arrow and hollow arrow in Figure 3A), tegmental area of the midbrain (arrow in Figure 3B), pons (hollow arrow in Figure 3C), and medulla (arrow in Figure 3D). As shown in Figure 3, the thalamic lesions had increased in size and new lesions had emerged (hollow arrow in Figures 3A,C). There were no evident lesions in the cervical and thoracic spinal cord at this stage (small arrows in Figure 3E). After 7 months, T2-weighted MRI (Figure 4) revealed hyperintensities in the bilateral thalamus (arrow in Figure 4A), tegmental area of the midbrain (arrow in Figure 4B), pons (arrow in Figure 4C), medulla (arrow in Figure 4D), and the cervical and thoracic spinal cord (new lesions, hollow arrow in Figure 4E). As shown in Figure 4, the previously identified lesions had decreased in size because of atrophy. After 8 months, T2-weighted MRI (Figure 5) revealed no new developments (arrow in Figures 5A–E).

Based on these results, the patient was treated with high-dose corticosteroids and immunoglobulin, although there was no obvious improvement in his severe respiratory insufficiency or limb weakness. Owing to the involvement of multiple systems and the patient's family history, we then suspected mitochondrial disease, following which muscle biopsy and gene detection studies were performed. Levetiracetam also substituted VPA for the treatment of epilepsy.

The patient experienced a gradual worsening of symptoms, developing myoclonus, ataxia, recurrent pneumonia, and hypotension. At this stage, he required sustained ventilator support and intermittent vasopressor treatment. Plasma lactate levels remained elevated. Neurological examination revealed somnolence, severe external ophthalmoplegia, severe limb weakness, myoclonus (evident in the proximal muscles of the upper extremities), ataxia, and pyramidal signs.

Treatment with levetiracetam, L-carnitine (2.0 g), coenzyme Q10 (CoQ10, 600 mg), nicotinamide, idebenone tablets, and vitamin B was initiated after obtaining the results of the genetic study. After 19 months, his symptoms had improved dramatically, and he required only non-invasive ventilator support during sleep. However, he died of recurrent pneumonia at the age of 18.

### Muscle biopsy

After 4 and 6 months of hospitalization, the patient underwent biopsy of the left quadriceps muscle. Serial frozen sections (thickness: 10 μm) were stained with modified Gomori trichrome (MGT), succinate dehydrogenase (SDH), and cytochrome C oxidase (COX), in accordance with established protocols (14).

**Figure 6:**
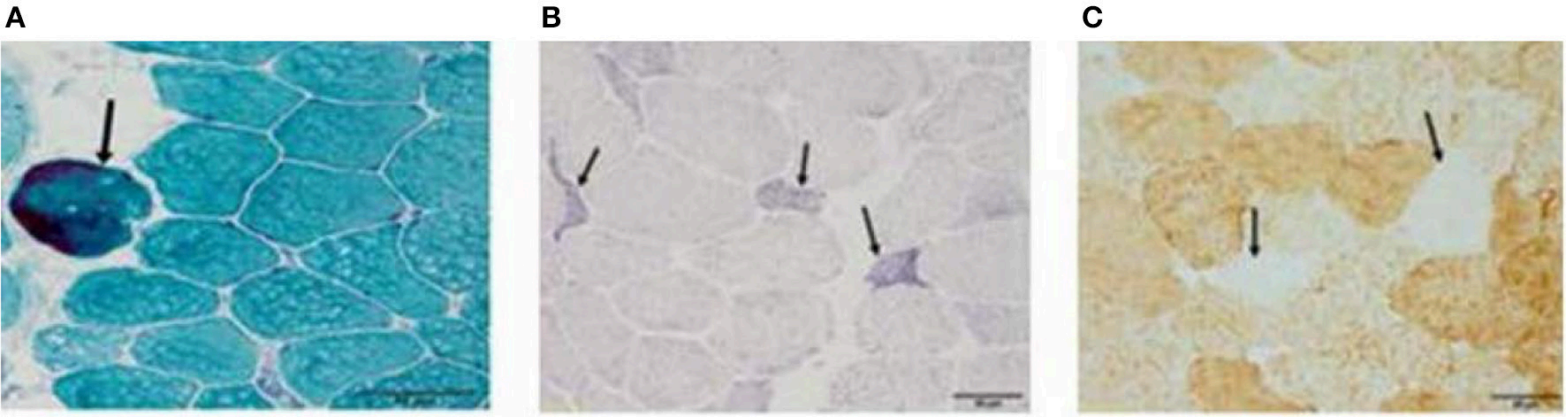
**(A–C)** After 4 months of hospitalization, muscle biopsy results revealed no remarkable mitochondrial abnormalities (including RRF) following histochemical analysis. However, 6 months after disease onset, modified Gomori trichrome staining of muscle biopsy specimens revealed the presence of scattered ragged red fibers (RRF, arrow in **A**), while succinate dehydrogenase staining revealed the presence of ragged blue fibers (arrow in **B**). Cytochrome C oxidase histochemical analyses revealed a diffuse reduction in the number of fibers (arrow in **C**). Bar represents 50 μm.

After 4 months of hospitalization, muscle biopsy results revealed no remarkable mitochondrial abnormalities (including RRF) following histochemical analysis. However, 6 months after hospitalization, MGT staining of muscle biopsy specimens revealed the presence of scattered RRF (arrow in Figure 6A), while SDH staining revealed the presence of ragged blue fibers (arrow in Figure 6B). Histochemical analyses after COX staining revealed a diffuse reduction in the number of fibers (arrow in Figure 6C). No excess lipid or glycogen levels were noted. Mitochondrial respiratory chain enzyme activity was not measured.

### Molecular genetic studies

Following hospitalization, total genomic DNA was extracted from the blood and the muscle in accordance with standard procedures. Long-range polymerase chain reaction (PCR) analyses were used to investigate potential rearrangements in the mtDNA, while direct sequencing of the entire mitochondrial genome was performed as previously described (15). Amplified PCR products were sequenced using Big Dye Terminator v3.1 (Applied Biosystems, Foster City, CA) and were compared to the revised Cambridge reference sequence (GenBank Accession number NC_012920.1). Quantification of m.8344A>G mutation load by pyrosequencing (16) was performed using mutation-specific primers.

**Figure 7:**
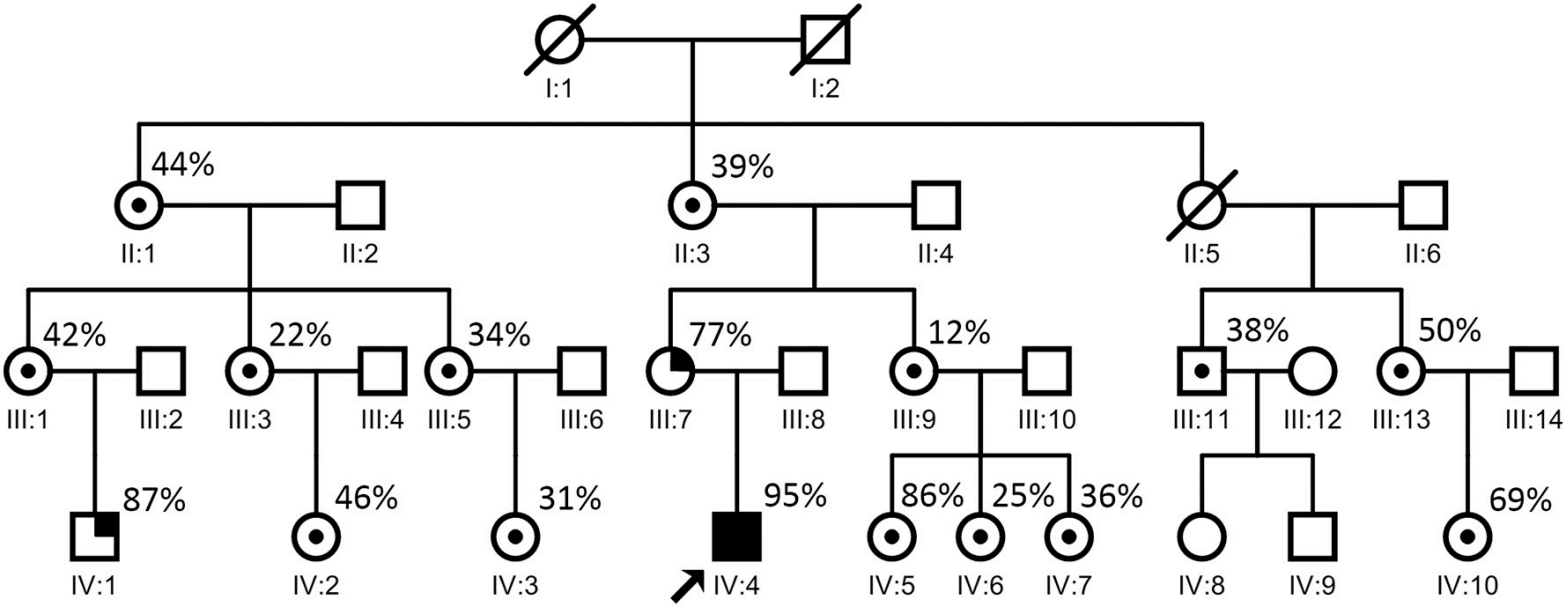
Proband's family pedigree. ○ = non-carrier, woman; □ = non-carrier, man; / = deceased; ⊙ = carrier; •■ = symptomatic members. The arrow indicates the proband. The patient's mother (III:7 in this figure) developed seizures at the age of 30. One of the patient's cousins (IV:1 in this figure) developed seizures at the age of 8. The point mutation level of these two relatives (from blood) were 77% (III:7 in this figure) and 87% (IV:1 in this figure), respectively, which are relatively high. Most of the other maternal relatives carrying the same mutation showed no evident symptoms.

Molecular analyses were conducted on muscle biopsy specimens from almost all of the patient's maternal relatives. Long-range PCR revealed no DNA rearrangements. Sequencing of the mitochondrial and nuclear genome in the blood and the muscle identified an m.8344A>G mutation. Quantitative pyrosequencing confirmed the presence of the m.8344A>G mutation at very high levels in the patient's blood (95%, IV:4 in Figure 7) and muscle (96%).

### Data of the affected family members

The patient's mother, who had short stature and increased resting and post-exercise blood lactate levels (2.7 and 9.7 mmol/L, respectively), developed seizures at the age of 30. One of the patient's cousins developed seizures at the age of 8. Seizures are one of the most common symptoms of mitochondrial diseases. The point mutation levels of these two relatives (from blood) were 77% (III:7 in Figure 7) and 87% (IV:1 in Figure 7), respectively, which were relatively high. Until the time of documentation, most of the other maternal relatives carrying the same mutation had shown no evident symptoms.

## Discussion

In the present report, we have discussed the case of a 16-year-old boy with a family history of epilepsy who presented with elevated lactate and glucose levels; bilateral, symmetrical, high-intensity signals in the thalamus, brainstem, and gray matter of the spinal cord on T2-weighted images; and RRF and COX-deficient fibers on muscle biopsy, all of which suggested that it was a mitochondrial disease. In fact, our patient fulfilled all the diagnostic criteria for Leigh syndrome (4, 17), including both typical neuroradiological and neuropathological features. In addition, he fulfilled the diagnostic criteria for MERRF (e.g., myoclonus, generalized seizures, ataxia, and RRF and COX-deficient fibers) (18), leading us to a diagnosis of overlapping Leigh syndrome/MERRF.

To date, only four cases of MERRF with Leigh syndrome and m.8344A>G mutation have been reported (19–22). Sweeney et al. reported the case of a 7-year-old boy with MERRF, ataxia, and myoclonic epilepsy who developed Leigh syndrome at the age of 18 when he contracted bacterial pneumonia (19). Laboratory analyses revealed that the patient also carried an m.8344A>G mutation (19). Among the 150 patients with m.8344A>G mutation, Silvestri et al. reported that only 2 patients developed Leigh syndrome. Although high mutation rates were detected in the muscle (100%) and lymphocytes (93%), detailed clinical information was not included in previous reports (20). Berkovic et al. (21) reported the case of a 4-year-old boy diagnosed with MERRF, who died at the age of 9 after contracting measles and pneumonia. Although brain autopsy results were indicative of Leigh syndrome, the authors did not investigate the rate of mtDNA mutation (21). Monden et al. reported the case of a 4-year-old boy diagnosed with MERRF who developed Leigh syndrome at the age of 6 (22). Genetic analyses revealed that the boy also had an m.8344A>G mutation (22). In three of these four cases, MERRF developed prior to the age of 7, with Leigh syndrome occurring 5–11 years later. However, our patient developed Leigh syndrome and MERRF almost simultaneously as an adolescent. To our knowledge, this is the first reported case of overlapping Leigh syndrome/MERRF in an adolescent patient. Consistent with the findings of Monden et al. (22), our patient also experienced recurrent pneumonia, which may have led to the deterioration of his condition.

Interestingly, almost all of the patient's maternal relatives (age range: 2–82 years) carried the same mutation, albeit at different rates (12–96%). The symptomatic mother exhibited seizures and high levels of blood lactate, with a 77% mutation rate in the muscle. One of his cousins, who exhibited an 87% mutation rate, also developed seizures. While all other relatives were asymptomatic, one of his cousins carried an 86% mutation, indicating that the mutation rate is not absolutely consistent with the presence of symptoms. These analyses, to our knowledge, represent the largest family pedigree associated with A8344G mutations in the mtDNA.

Notably, our patient also developed repeated hypotension, although heart function and fluid volume remained normal, necessitating intermittent treatment with vasopressors. To our knowledge, no such findings have been described in previous reports. We speculate that the hypotension in our patient may have been due to autonomic nerve dysfunction.

The differential diagnosis included the non-alcoholic variant of Wernicke's encephalopathy, although this was easily excluded.

In conclusion, the present report is the first to describe a case of overlapping Leigh syndrome/MERRF in an adolescent patient using a large pedigree of the mtDNA A8344G mutation, further expanding the clinical spectrum associated with the m.8344A>G mutation. Our findings also emphasize the significance of comprehensive clinical features, imaging, histology, and genetic studies in the diagnosis of mitochondrial diseases. In future studies, we intend to examine the full pedigree of the patient's family.

## Patient consent

Consent was obtained for publication of the case details.

## Ethics statement

This present study was approved by the Ethics Committee of the Sun Yat-Sen University.

## Author contributions

CS conceived, designed, and performed the experiments, designed the data collection tools, monitored data collection for the whole trial, processed and analyzed the data, and drafted and revised the manuscript. WX monitored data collection for the whole trial, analyzed the data, and drafted and revised the manuscript. LC, XiL, XuL, and HZ conceived and designed the experiment, revised the manuscript, approved the final version, and were responsible for the overall content as guarantors.

### Conflict of interest statement

The authors declare that the research was conducted in the absence of any commercial or financial relationships that could be construed as a potential conflict of interest.
